# It takes more than two to tango: mechanosignaling of the endothelial surface

**DOI:** 10.1007/s00424-020-02369-2

**Published:** 2020-04-01

**Authors:** Benedikt Fels, Kristina Kusche-Vihrog

**Affiliations:** Institute of Physiology, University of Luebeck, Ratzeburger Allee 160, D-23562 Lübeck, Germany

**Keywords:** Mechanosensitive ion channels, Glycocalyx, Mechanotransduction, Shear stress sensor, Nanomechanics

## Abstract

The endothelial surface is a highly flexible signaling hub which is able to sense the hemodynamic forces of the streaming blood. The subsequent mechanosignaling is basically mediated by specific structures, like the endothelial glycocalyx building the top surface layer of endothelial cells as well as mechanosensitive ion channels within the endothelial plasma membrane. The mechanical properties of the endothelial cell surface are characterized by the dynamics of cytoskeletal proteins and play a key role in the process of signal transmission from the outside (lumen of the blood vessel) to the interior of the cell. Thus, the cell mechanics directly interact with the function of mechanosensitive structures and ion channels. To precisely maintain the vascular tone, a coordinated functional interdependency between endothelial cells and vascular smooth muscle cells is necessary. This is given by the fact that mechanosensitive ion channels are expressed in both cell types and that signals are transmitted via autocrine/paracrine mechanisms from layer to layer. Thus, the outer layer of the endothelial cells can be seen as important functional mechanosensitive and reactive cellular compartment. This review aims to describe the known mechanosensitive structures of the vessel building a bridge between the important role of physiological mechanosignaling and the proper vascular function. Since mutations and dysfunction of mechanosensitive proteins are linked to vascular pathologies such as hypertension, they play a potent role in the field of channelopathies and mechanomedicine.

## Introduction

Maintaining vascular homeostasis and keeping blood pressure variations in an optimal physiological range are a lifelong challenge which among others ensure a sufficient blood flow and supply of oxygen and nutrients to peripheral organs. Therefore, pump function of the heart, vascular resistance and renal water, and salt homeostasis are closely monitored by various physiological mechanisms, which reconcile metabolic demand and supply on an acute and long-term scale. Endothelial cells (EC) are located at the innermost layer of all blood and lymphatic vessels. They are constantly exposed to mechanical forces mediated by the blood flow, thereby maintaining a selective permeable barrier between the tissue and intravascular lumen. In addition to this transport barrier, EC contribute to the regulation of blood pressure and represent a multifunctional signal-transducing surface. EC function can thereby be modified by a bench of biochemical signals (catecholamines, neurotransmitter, cytokines, growth factors) [100, 173, 186, 196] as well as mechanical stimuli coming from the blood stream itself [38, 72, 134]. Blood flow induced hemodynamic forces such as shear stress, hydrostatic pressure, and circumferential stretch can be sensed by EC through mechanosensors and transferred into signaling pathways, modifying gene and protein expression and endothelial function [113].

The different hemodynamic forces vary depending on, e.g., physical activity, different vessels types, vessel location (bifurcation sites), and—temporally—on the pulsatile cardiac action. Even at the level of EC, there is a distinct spatial distribution of the external forces acting on different cellular mechanosensors. These mechanical forces are sensed and translated into biochemical signals by specific structures and proteins located in the membranes of endothelial cells. During the last years, a number of potential cellular mechanosensitive and responsive structures have been identified so far, including cell adhesion proteins (like VE-Cadherin, PECAM-1), ion channels, tyrosine kinase receptors (VEGF receptor 2), G-protein coupled receptors (GPCR), caveolae, primary cilia, cytoskeletal actin, nesprins, integrins, and the endothelial glycocalyx (eGC) [43, 81, 193].

After being sensed by the EC, the mechanical forces are encoded and transmitted to the vascular smooth muscle cells (VSMC), which either respond with relaxation or contraction. In fact, a close functional interaction between EC and the neighboring VSMC is responsible for the regulation of the vascular tone and the ability of cells to react on different biochemical and mechanical stimuli from the streaming blood. During the last years, it became clear that in particular the mechanical properties of EC (i) depend on flow-mediated forces and (ii) determine the contraction status of VSMC. This well-described mechanism is mainly based on the ability of the EC to release nitric oxide (NO) in a shear stress-dependent manner, which diffuses to adjacent VSMCs where it triggers vasodilation via cGMP-dependent pathways [154]. A reduction in NO is strongly associated with increased levels of reactive oxygen species (ROS) generated by NAD(P)H oxidase, xanthine oxidase, or uncoupled endothelial nitric oxide synthase (eNOS) within the vascular wall, leading not only to scavenging of NO but also to disruption of some signaling pathways that mediate its production [16]. Hence, the tight interplay between EC and VSMC controls vascular function and vessel tone. Primarily the ability of the EC to change their mechanical properties, i.e., to alternate between “stiff” and “soft” conditions, is an important physiological feature of the endothelium. Endothelial cells which have lost this ability and are arrested in chronic stiffening can be seen as dysfunctional [95].

This review mainly focuses on the impact of the endothelial glycocalyx and mechanosensitive ion channels in endothelial mechanosensing. The endothelial cell surface, including glycocalyx, plasma membrane, cortex, and ion channels, can be seen in total as important functional mechanosensitive and reactive cellular compartment. Since mutations and dysfunction of mechanosensitive structures are linked to vascular pathologies such as hypertension [83, 127, 169], they play a potent role in the field of channelopathies and mechanomedicine.

### Shear stress-mediated mechanosignaling

Due to their position, EC sense and react to changes in shear stress caused by the blood stream, which is substantial for a proper physiological vascular function [5, 31]. It is generally accepted that shear forces lead to an EC-mediated vasodilation due to secretion of vasoactive substances like NO [154]. Other known shear stress-induced mediators involved in the control of vascular tone are prostacyclin, a potent vasodilator [18, 111, 139], and endothelin, a strong vasoconstrictor and different mitogenic molecules [198]. This vasomodulatory secretion can mediate increase as well as decrease in vessel diameter. This mechanism is also known as flow-mediated dilation (FMD) and its impairment, caused on a decreased NO production, which can be seen as a hallmark of endothelial dysfunction [46, 55, 95]. In line with this, FMD was found to be markedly reduced in hypertensive patients and diseases like hyperaldosteronism [129, 138].

Shear stress is a tangential force arising due to the friction of the blood volume and the vessel wall (in fact the EC). It varies over the vascular tree from 1 dyne/cm^2^ at venous ECs up to 40 dyne/cm^2^ in arterial vessels [87, 193]. Another type of force is the blood pressure itself, exerting a variable magnitude, ranging from 120 to almost 0 mmHg (MAP; mean arterial pressure) depending on different types and location of the blood vessels. Both forces mediate the third type of force, the so-called circumferential stretch, acting through transmural pressure differences distending the vessel wall [144]. EC response to hemodynamical variations of the blood flow ranges from acute adaptations in ion channel function to long-lasting gene regulatory events [33, 67]. EC can also respond to shear stress with cytoskeletal remodeling by increasing actin stress fibers [14]. Here, it is important to differentiate between laminar and non-laminar (turbulent) forms of shear stress [98], since these different forms of shear stress modulate many different effects in the vascular system. Laminar shear stress (LSS) physiologically occurs mainly at straight parts of the blood vessels and is known to mediate protective properties such as down-regulation of inflammatory cytokines, adhesion molecules, and oxidative stress [69, 76, 102]. These positive effects are mainly caused by the physical properties of LSS as an ideal-typical parabola shape flow, where the shear rate is decreased at the center of the lumen of the blood vessel and gradually increased toward the wall [18]. Disturbances of the hemodynamic homeostasis are associated with cardiovascular diseases [22]. Especially, pathologic changes in the rheology of the blood lead to and maintain atherogenic processes – especially in the branching regions of blood vessels where non-laminar shear stress (NLSS) occurs.

In contrast to LSS, NLSS is defined as the flow in which the blood velocity varies continuously over the course of time, even though the overall flow may remain steady [18]. This can explain the pathophysiology of atherosclerotic lesion, which non-random distribution can be attributed to the alterations of local function of vascular ECs by a disturbed flow pattern like flow separation, recirculation, reattachment, low and reciprocal shear stress, and high spatial and temporal gradients of shear stress [22].

**Fig. 1:**
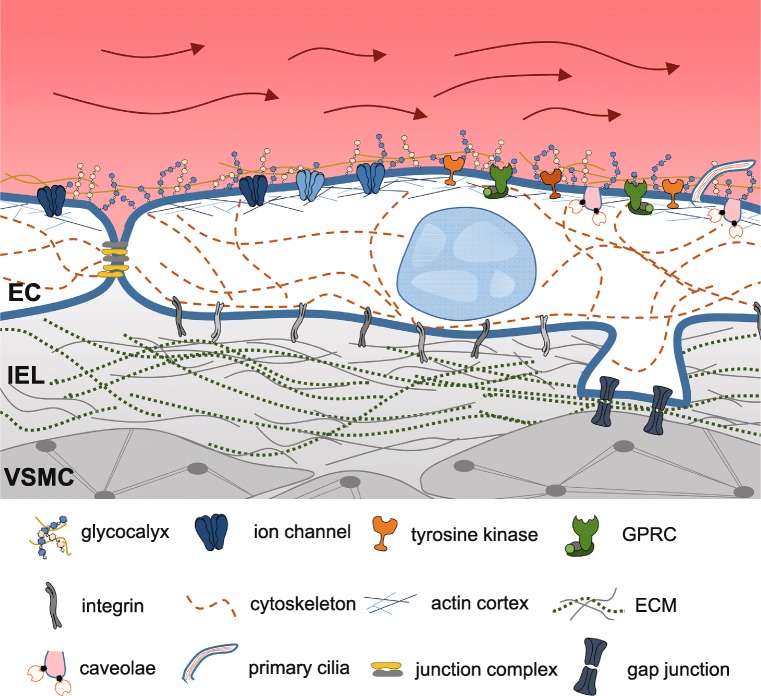
Mechanosensitive structures of the endothelium. Blood flow-induced hemodynamic forces such as shear stress, hydrostatic pressure, and circumferential stretch can be sensed by EC through mechanosensors. These structures sense the mechanical forces and translate them to biochemical signals by specific proteins located on/in the membranes of endothelial cells. Potential cellular mechanosensitive and responsive structures are depicted in this figure. *EC*, endothelial cell; *IEL*, internal elastic lamina; *VSMC*, vascular smooth muscle cell

To understand these flow-mediated alterations in EC function and dysfunction, a detailed knowledge of EC mechanosignaling is crucial. The following chapters will focus on different mechanosensitive structures within the endothelium (see Fig. 1 for overview).

### Mechanosensitive structures in the endothelium

The *cellular tensegrity model* has been proposed to explain transduction of mechanical forces to biochemical signals [79]. It is based on the concept that complementary mechanical forces arising from the cytoskeleton and extracellular tethering sites to the ECM or neighboring cells are balanced. A shift in this equilibrium mediates mechanosensing and signal transduction [80]. Based on a cellular level, a hierarchical and multi-modular tensegrity structure is postulated. In line with this model, traction force microscopy analyzes cell tension (= cell adhesion) exerted from cytoskeletal parts to its anchoring points of the ECM on a flexible polyacrylamide substrate [137]. Sims and colleagues were able to show that EC exert force to the substrate which can be revoked by trypsin treatment [160]. Individual stress fibers are tensed by actomyosin motors and confer the forces to the ECM, thereby modulating a cellular pre-stress which is transmitted to and balanced by traction forces that act at the cell-anchoring points to the substrate [80, 93].

*Tyrosine kinase receptors* (e.g., VEGFR2 or Tie-2) are activated in ECs after shear stress exposure in a ligand-independent manner [85, 97, 176]. The mechanism which leads to phosphorylation of VEGFR2 in response to shear is still not well understood. VEGFR2 seems to work in a network along with PECAM-1 and VE-cadherin, mediating the intramembrane binding to the whole mechanosensory complex [27]. The eGC could be identified as another interaction partner of VEGFR2, thereby regulating receptor endocytosis and activation in response to eGC composition [96]. Of note, the endothelium-stabilizing receptor Tie-2 was found to be deactivated during sepsis, leading to an eGC breakdown, and could be prevented by Tie-2 activation and blockage of Tie-2 antagonist angiopoietin [40].

*GPCR and G-proteins* have been identified in shear stress signal transduction in various studies. For example, GPR68 could be identified in a shear stress RNAi library screen as a necessary component for flow-mediated dilation in small resistance arteries [192]. Additionally, G-proteins can be activated by shear stress independently from GPRC activation. The G-protein Gα_q/11_, for example, could be activated by shear in the presence of GPCR antagonists in the human coronary artery endothelial cells [36].

Caveolae, small cholesterol and glycosphingolipid-rich flask-shaped membrane invagination, form membrane microdomains containing various signaling molecules, including the aforementioned kinases, GPCR, and ion channels [15, 54, 157]. eNOS is associated with caveolae and its positioning is coupled to proper NO production [61, 156]. Redistribution of eNOS away from the plasma membrane depends on cholesterol composition of the caveolae. Oxidized low-density lipid and cholesterol depletion lead to reduce NO production [177]. Proteins within the caveolae like caveolin-1 thereby inhibit eNOS function and participate in EC-mediated vasodilation [19]. In addition, caveolin-1 stabilizes eNOS expression level and is proposed to be an important determinant of endothelial vasodilatory functions [20].

The endothelial barrier is formed by tight junctions, VE-cadherin and PECAM-1 [176]. The vascular permeability is thereby mainly controlled by VE-cadherin in a Ca^2+^-dependent manner [35]. Cadherin complexes are connected to the cytoskeleton via catenin and vinculin and can remodel in response to mechanical stimuli [75]. Activation of vinculin can lead to F-actin polymerization, and VE-cadherin and PECAM-1 protein complexes can be altered in response to shear stress [26, 172]. Cell-matrix interactions via *integrins* are also discussed to be part of the mechanosensitive complex in EC [21]. However, evidences for a direct activation of integrins by shear stress are limited. Integrins more likely are activated by biochemical and not force-based signals arising from other primary mechanosensors [105, 174].

*Primary, non-motile cilia* are protrusions of the apical cell membrane with an extend up to 5 μm and consist of microtubule bundles, which are connected to the intracellular cytoskeleton [45]. Cilia are sensitive to shear stress and can be disassembled by LSS (15 dyne/cm^2^), accompanied by major rearrangement of the cytoskeleton [82]. Cilia mediated shear stress sensing coupled to Ca^2+^ signaling and nitric oxide production. Knockdown of cilia proteins lead to disturbed mechanosignaling [125, 126].

The *cytoskeleton* is composed of three major filament types, namely (i) the microfilaments, (ii) intermediate filaments, and (iii) microtubules. This cytoskeletal scaffold can be deformed and transmits force/tension via focal adhesion sites, integrins, cellular junctions, and extracellular matrix to the interior of the cell [32]. Microfilaments consist of actin polymers, which can be rearranged highly dynamically by change from filamentous actin (F-actin) to globular actin (G-actin) and can be connected between cellular structures. De/stabilization is mainly mediated by members of the Rho family of small GTP-binding proteins like Rho and Rac GTPases. To counteract external tensile forces, actin can polymerize in response to tensile forces, leading to stress fiber formation, which are composed of actin and myosin II filaments [71, 132, 178].

*Intermediate filament* proteins like laminin form the nuclear scaffold adjacent to the inner nuclear membrane. It thereby contributes to chromatin regulation and signaling pathways affecting gene expression [84]. It is discussed, that laminins act as a “mechanostat” that is able to sense extracellular forces and respond by reinforcing the cytoskeleton and the extracellular matrix, e.g., by directly transducing external forces to the nucleus which alters gene expression [123, 135]. Microtubules are involved in shear stress-derived cell polarity and are interconnected as well as linked to membrane proteins throughout the cell [175, 176]. Recently, it could be shown that microtubules also interact with integrin-based focal adhesions and myosin IIA filaments [141]. This connection of external contact, adhesion receptors, and cytoskeletal structures serves as a potent mechanotransducer for inside-out as well as outside-in signaling pathways.

The following chapters will mainly focus on the impact of the *endothelial glycocalyx and connected mechanosensitive ion channels* in the vascular mechanosensing. Being mechanosensitive switches, ion channels convert mechanical stimuli attaining the cell membrane (pressure, stretch, shear) into electrical and biochemical signals, which affect the cellular and physiological reactions.

### Endothelial glycocalyx

The glycocalyx is the top surface layer of all living cells, including endothelial cells, and is built by a negatively charged, brush-like structure, with a functional height up to 500 nm. This membrane-bound carbohydrate-rich layer covers the luminal membrane of endothelial cells (endothelial glycocalyx, eGC) and is associated with different plasma proteins [187]. Together with the cortical actin, a thin actin mesh directly underneath the plasma membrane, and membrane proteins, like mechanosensitive ion channels, the eGC build a highly dynamic hub for intra- and extracellular signals [52, 88]. eGC functions range from modulation of leukocyte adhesion, regulation of blood coagulation, maintaining vascular permeability barrier, and mediating flow-induced NO release. So, it has been recognized as an important vasculoprotective nanobarrier [28, 64]. For a detailed overview of the eGC nanomechanics and functions, we refer to a recent review from our group [28].

The eGC is formed by glycoproteins and proteoglycans like heparan and chondroitin sulfate as well as hydrophilic hyaluronic acid [146, 151, 170]. The components are covalently anchored, and transmembrane proteins like syndecan link the eGC with the intracellular actin cortex [143]. This enables the eGC to transduce extracellular signals into intracellular biochemical signaling pathways. In the same time, because of its intrinsic charge, other negatively charged molecules (or cells) from the plasma are hindered from passing this first barrier [25]. From this point of view, the eGC acts as an effective cation buffering and barrier system [41, 153]. Under healthy physiological conditions, the eGC structure is in a steady state of permanent turnover caused by flow-mediated degradation and reorganization by biosynthesis of new eGC components. The exact turnover of eGC can hardly be analyzed, known values range from 6 h in enterocytes to 5 days in rat uterine epithelial cells [58, 86]. However, eGC must be seen as a highly flexible and inhomogeneous structure in dependence of EC (and eGC) positioning along the vessel tree as well as due to various electrostatic and biochemical interactions between its constituents [121, 189].

### eGC as mechanosensor

Due to its unique localization as an interface between the blood stream and tissue, the eGC has been identified to function as a mechanosensor as well as mechanotransducer [6, 166, 187]. For example, Yen and colleagues showed that flow-induced NO production in post-capillary venules and arterioles of rat mesenteric arteries can be abolished by enzymatic removal of heparan sulfate by heparanase III treatment. The authors postulate that the eGC acts as a mechanotransducer and participates in the regulation of NO production [194]. Dragovich and colleagues showed in brain microvascular endothelial cells that enzymatic removal of eGC components lead to perpetuated Ca^2+^ signals and eNOS activity [39] accompanied by the remodeling of cytoskeletal structures [3]. In fact, the eGC itself can be modulated in structure and function in response to changes in blood flow [68, 187]. Shear stress induces remodeling of the eGC, by increasing heparan sulfate, chondroitin sulfate, glypican-1, and syndecan-1 at the cell surface, thereby influencing the integrity of the glycocalyx and its ability of sensing shear stress [195]. In addition, shear stress acting on the EC stabilizes the eGC, which is important for proper endothelial function and NO production [168, 194]. For example, laminar shear stress induces a recruitment of hyaluronan synthase 2 to the endothelial plasma membrane and increases hyaluronan expression, a major structural eGC component [184]. In line with this, the presence of heparan sulfate, and thus an intact eGC, is necessary for flow-induced NO production in aortic EC [57]. eGC breakdown by antagonism of endothelium-stabilizing receptor Tie-2 leads to plasma leakage and increased leukocyte recruitment in vivo [109].

These findings strengthen the idea of a vasculoprotective function of the eGC [64, 189]. However, it is postulated that stabilization and turnover of the eGC by shear stress might rather be a physiological response to mechanotransductory changes under flow conditions. We were able to show that moderate laminar shear stress (LSS, 8 dyne/cm^2^) increased the amount of heparan sulfate at the surface of endothelial cells, while treatment with heparanase I leads to a significant reduction of the eGC under shear stress conditions. Delgadillo and colleagues also showed shear-mediated effect on the physical nanobarrier function of eGC. Comparisons of different shear rates on HUVECs lead to higher eGC thickness and decreased neutrophil adhesion under high (10 dyne/cm^2^) vs. low (0.5 dyne/cm^2^) shear stress [37]. In addition, moderate LSS leads to increase F-actin polymerization within the actin cortex (unpublished data of our group). This illustrates that physiological shear stress is obligatory for a proper eGC structure and plasticity to fulfill mechanosensory function within the vascular system.

As described above, from the pathophysiological point of view, a damaged eGC exerts a disturbed mechanotransduction to intracellular components like the endothelial actin cortex and will change membrane characteristics including the presence of mechanosensitive ion channel, adhesion molecules, and cytoskeletal anchor proteins [197]. Different authors postulate feedback reinforcement between damaged eGC and progression of endothelial dysfunction [48, 159, 197].

First observation of a pathophysiological damage of eGC was done by Van den Berg. He screened atheroprone regions of mouse internal carotid arteries and observed a reduced eGC thickness in disease predilection compared with common carotid arteries [179]. Others found higher eGC component synthesis (because of higher eGC turnover) in arteries exposed to higher shear stress compared with low shear stress [64]. Different non-cardiovascular as well as cardiovascular diseases are accompanied by disturbed eGC mechanosensing. In an in vitro model of hyperglycemia, a disturbed flow-mediated alignment of EC was accompanied by loss of heparan sulfate (major eGC component), as well as reduced NO production in response to shear stress application [17, 108]. Dialysis patients show impaired eGC structure and shedded hyaluronic acid as well as syndecan-1 in the blood [180]. Also the impact of eGC damage on glomerulus filtration and development of albuminuria are widely discussed [149]. High ox-LDL levels induce degradation of the eGC and subsequently increased leukocyte adhesion in cremaster venules [25]. Knockdown of syndecan-1, an important eGC component, lead to impaired mechanosignaling in injured carotid arteries, larger neointimal hyperplasia, and increased VSMC proliferation [59]. Lack of syndecan-1 is associated with impaired migration and enhanced adhesion of macrophages, as well as increased inflammation and atherosclerotic plaque formation [4].

In conclusion, disturbance of the eGC structure, e.g., by changes in blood flow parameters lead to altered mechanosignaling in EC. These results strengthen the concept of a mutually interacting signaling hub of eGC, cortical actin, and ion channel within the endothelial cell.

### Mechanosensitive channels in the endothelium

In response to shear stress or flow-mediated membrane stretch, opening of mechanosensitive ion channels is the very first step in cellular mechanosignaling [24, 115, 122]. These ion channels show partially opposed characteristics, ranging from hyperpolarization by K^+^ selective TREK channels to depolarization by Ca^2+^ and Na^+^ permeable Piezo1 channels. Here, we mainly focus on mechanosensitive cation permeable ion channels, leading to Ca^2+^ influx into the cell. There is substantial evidence that the increase in intracellular Ca^2+^ is one of the earliest events in response to shear stress. In endothelium, increased Ca^2+^ subsequently activate eNOS and intermediate conductance Ca^2+^-activated K^+^ channels (IK_Ca_), resulting in vasodilation through eNOS-mediated NO release and/or membrane hyperpolarization. Ion channels seem particularly well suited to perceive physical forces and are strongly suggested as key players in the sensing of shear stress [77].

*Piezo1 and Piezo2* are mechanically activated cation channels which mediate as large homomultimeric complexes cation currents in various tissues [29, 181]. Both isoforms are mechanically gated and confer nonselective (Na^+^, K^+^, and Ca^2+^) currents with fast activation kinetics. Whereas Piezo2 is mainly expressed in tactile epithelial cells (Merkel) [190] and mechanosensory neurons [122], Piezo1 has been reported to mediates mechanically induces currents in various cell types, including endothelial cells and smooth muscle cells [142, 145]. Piezo1 was shown to be an important sensor of shear force in EC and involved in cell alignment in flow direction [103]. Laminar flow-mediated activation of Piezo1 mediates flow-induced release of ATP from endothelial cells, resulting in the activation of the G_q_/G_11_-coupled purinergic P2Y_2_ receptor [182, 183]. P2Y_2_/receptor and G_q_/G_11_ cascades lead to activation of AKT and eNOS and mediate flow-induced vasodilation. Both laminar and disturbed flows activate the same initial mechanosignaling pathway involving Piezo1- and G_q_/G_11_-mediated signaling [2]. Accordingly, Piezo1 channel activator Yoda1 induces NO-mediated relaxation of murine intrapulmonary arteries [101].

*Transient receptor potential (TRP)* channels are non-voltage gated cation channels, regulated by polymodal stimuli and implicated in a variety of cellular functions [128]. At least ten TRP channels (TRPC1, 5, 6; TRPV1, 2, 4; TRPM3, 7; TRPA1; TRPP2) have been proposed to be mechanosensitive [24, 81, 112, 158, 171]. TRP can mediate Ca^2+^ signaling but also can be Ca^2+^ regulated by directly Ca^2+^ binding to the channel or Ca^2+^-calmodulin complex mediated activation. In VSMC, Ca^2+^ influx through TRP channels leads to membrane depolarization and forced influx through voltage-gated Ca^2+^ channels (L-type or T-type Ca^2+^ channels, Ca_V_1.2 / Ca_V_3.1). Ca^2+^-calmodulin complex activates myosin light chain kinase and initiates the contractile process [73].

In many cases, it is still not finally clarified how this mechanosensivity is mediated, although a number of studies support the mechanosensitive characteristics of TRP channels [73]. The following principles are discussed: (i) direct activation by extracellular forces like membrane stretch and shear-induced changes in lipid bilayer conformation and subsequent deformation of channel domains [60, 114, 162], (ii) tethering of ion channel structures with cellular component like ECM, proteins, or intracellular cytoskeleton [9, 117], and (iii) indirect activation by other primary mechanosensors and subsequent biochemical transduction to effector TRP channels [91, 107, 119]. In the following sections, some mechanosensitive candidates of the TRP family will be discussed.

*TRPV4* has been proposed to be a candidate for the molecular blood flow sensor inducing the flow-induced vasodilation, a response to increased blood flow velocity or viscosity [31]. Consistently, TRPV4 was identified to be activated under hypertonic conditions and cell swelling-mediated membrane stretch [104]. On the other hand, cell-attached patch clamp approaches were not able to directly activate TRPV4 by pipette suction, suggesting an indirect activation of TRPV4 through force-sensitive signaling cascades [43]. Kohler and colleagues showed that in rat carotid artery, endothelial cells agonist- or shear stress-induced activation of TRPV4 leads to dilation of rat gracilis arteries. eNOS blockade attenuates this TRPV4-mediated effect [91]. The same group was able to show that TRPV4 knockout showed significantly reduced flow-induced vasodilation [70]. In line with this, Mendoza and colleagues found a TRPV4-dependent relaxation involving NO and EDHFs and Ca^2+^ influx through endothelial TRPV4 channels in response to flow [120]. Shear stress also leads to exocytosis-mediated recruitment of TRPV4 channels and endothelial sensitization to mechanical stress [8].

TRPV4 was found to be co-localized with TRPC1 proteins in EC from rabbit mesenteric arteries. Analysis of (high external) Ca^2+^-induced EC-dependent vasodilation showed TRPV4- and TRPC1-dependent Ca^2+^-influx and induction of NO production. Activation of TRPV4 (agonist) induced NO production, and subsequent vasodilation could be prevented by L-NAME (N(ω)-nitro-L-arginine methyl ester, eNOS inhibitor), TRPV4 antagonist (RN1734), or TRPC1 antagonism (T1E3, blocking peptide). Heteromeric TRPV4 and TRPC1 channels mediate calcium-sensing receptor induced vasorelaxation through NO production [65].

The *TRPC1* channel is the first cloned member of the mammalian TRP superfamily [188]. TRPC1 function is generally associated with regulation of store-operated Ca^2+^ channels (SOC) and receptor-operated Ca^2+^ channels (ROCC) via interaction with STIM1, ORAI1, and IP3 receptors [10, 34]. Mechanical (tonic) stretch application for 14 h to human myometrial smooth muscle cells leads to increased expression (qPCR and WB) of TRPC3 and C4, but not of TRPC1 or C6 [30]. On the other hand, up-regulation of TRPC1, C3, and C6 could be found in pressurized hearts after aortic constriction, suggesting mechano-responsive expression pattern of TRPC1 channels [94, 133]. Nevertheless, TRPC1 as mechano-sensitive channel has been a subject of controversial debates [11, 63]. Overexpression of TRPC1 in frog oocytes increased the number of stretch-activated ion channels in patch clamp experiments, which can be diminished by siRNA approach [114]. In cancer-associated fibroblasts, TRPC1 is involved in responding to an increase of the ambient pressure [53], whereas in MDCK-F cells, TRPC1 also contributes to mechano-signaling during cell migration [47]. TRPC1 has also been identified as a component of biomechanical signaling in the development of pressure-induced heart failure and hypertrophy [44, 155].

It is controversially discussed if TRPC1 acts as homomeric or at least as a heteromeric channel together with TRPC3/4/5 or TRPV4 [63, 110, 163, 165]. In HUVECs (primary human umbilical vein endothelial cells), agonist-mediated stimulation of calcium-sensing receptor (CaSR) leads to a TRPC1-dependent increase in intracellular Ca^2+^ and enhances NO production. The authors postulate a coupling of TRPC1 to CaSR and TRPC1-mediated store-operated Ca^2+^ entry (SOCE) mechanisms for Ca^2+^ influx [140]. TRPC1 is also co-localized with TRPV4 in EC from mesenteric arteries. This heteromeric channel is activated by CaSR and induces an increase in NO production and vasorelaxation [65, 66].

*TRPC6* is potentially a mechanosensitive TRP channel, which can be activated directly by diacylglycerol (DAG) [74, 92]. TRPC6 is important for regulating endothelial permeability in response to pro-inflammatory cytokines and inflammatory markers [99, 161]. In EC of the pulmonary arteries, TRPC6 knockout diminished the TRPC6 agonist-mediated increase in intracellular Ca^2+^, vascular filtration, and edema formation [150]. Fleming and colleagues showed that cytochrome P450 (CYP)-derived epoxyeicosatrienoic acids (EETs), one amongst other mechanically produced metabolites, supports translocation of TRPC6 to caveolin-1-rich cell membrane areas [56]. The direct mechanical activation of TRPC6 is discussed controversially. Inoue et al. proposed a synergistic activation by a combined mechanical and muscarinic receptor agonist carbachol-mediated stimulation [148].

*TRPM7* expression could be shown in HUVECs by Baldoli and colleagues [7], where it has been linked to magnesium transport. TRPM7 is somehow unique in comparison with other TRP because it possesses a regulatory kinase domain at the C-terminus [147]. The mechanosensitive potential of TRPM7 could be shown in pressure-loading patch clamp approaches [191] as well as in fluid shear stress experiments in mesenchymal stromal cells [106].

*TRPP2* (also known as polycystin-2 and polycystic kidney disease 2, PKD2) has been linked to mechanosensitive functions of primary cilia. Reduced expression of TRPP2 leads to decreased NO production in murine EC [1]. Knockdown of TRPP2 leads to an inability of EC to transduce extracellular shear stress into intracellular Ca^2+^ signaling and biochemical nitric oxide synthesis [125]. Also an interaction between TRPP2 and TRPC1 and a potential role in stretch-induced injury of blood-brain barrier endothelial cells is postulated [12, 136]. Additionally, it was observed that only a heteromeric channel composition of TRPP2, TRPC1, and TRPV4 is able to mediate flow-induced cation currents [42].

The epithelial sodium channel *ENaC* has primarily been described in principal cells of the distal nephron in the kidney, where it is mainly involved in salt and water homeostasis [13, 62]. Now it is obvious that ENaC is expressed in a variety of different tissues where it fulfills diverse functions. In particular, ENaC was identified in the vascular endothelium, where it controls endothelial nanomechanics [50, 167]. ENaC, like many other ion channels, is linked to cytoskeletal components and these interactions are used for mechanotransduction [51, 78, 118, 185]. It is proposed that an increase in ENaC activity in EC and thus an enhanced sodium influx stabilizes cortical actin in its filamentous form (F-actin), leading to a more rigid cell cortex [131, 185]. Unpublished data from our group show that functional inhibition of ENaC provokes a shift from F- to G-actin which in turn leads to a softening of the cell cortex. In contrast, chemical stabilization of the actin cytoskeleton abrogates this effect. Hence, ENaC function and actin dynamics are strongly correlated in EC.

In the case of the epithelial ENaC, a flow-modulated stimulation of ENaC activity and sodium absorption is mediated by an increase in hydrostatic pressure, suggesting a flow-sensitive way of channel gating [152]. In addition, Guo and colleagues showed that ENaC can be activated by flow and increased hydrostatic pressure, and increased intracellular sodium levels lead to reduce NO production in EC [67]. In line with these findings, we were able to show that ENaC is inserted into the membrane in response to acute shear stress modulations (unpublished data from our group). This leads to increase Na^+^ influx into the EC and polymerization of the cortical actin. A recent publication shows that ENaC shear force sensing is dependent on sugar residue interaction with the eGC. Extracellular N-glycosylated asparagine residues of ENaC interconnect the channel with the ECM as well as eGC, and removal of these N-glycans lead to decreased shear force-induced ENaC currents [90]. These data support the idea of a tight interaction and interdependence of eGC, ion channel function, and cytoskeleton as coupled mechanosensors of the endothelium.

### Interaction between mechanosensitive ion channels in the VSMC and EC

**Fig. 2:**
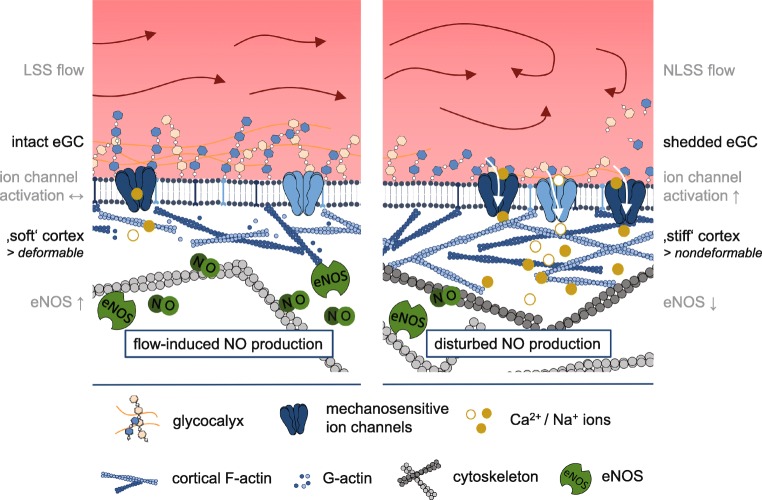
Model of eGC- and ion channel-mediated mechanosignaling. Physiological LSS is accompanied by an intact eGC structure and a “soft” and deformable actin cortex. EC can react to changes in blood flow with increased eNOS activity and NO-mediated vasodilation (left figure). Pathophysiological increase of shear stress (e.g., by NLSS) leads to a disturbed eGC structure, increased Ca^2+^, and Na^+^ influx and stiffening of the cell cortex. This is accompanied by reduced eNOS activity and impaired flow-mediated vasodilation (right figure). The ability of the EC to change their mechanical properties, i.e., to alternate between “stiff” and “soft” conditions, is an important physiological feature. Loss of this plasticity leads to a dysfunctional endothelium

The regulation of the vascular tone is basically mediated by processes within the vessel wall. As mentioned before, EC and VSMC are in close physical vicinity and their functions are tightly coupled. Hence, biochemical as well as mechanical signals from the streaming blood are recognized by the endothelial surface structures and conducted to the VSMC. One of the best described paracrine mechanism of EC-VSMC interplay is the EC-derived NO release which directly affects the contraction status of the VSMC: A high production of NO in EC leads to relaxation of the VSMC and decreased vessel tone, while a reduction of NO release causes contraction of the VSMC and increased vessel tone. This in turn is directly linked to the mechanical properties of endothelial cells: A soft endothelial cell cortex is easily deformable by the streaming blood and thus the endothelial cell releases higher amounts of NO in contrast to a stiff cell cortex [51, 130]. The mechanical properties of the endothelial surface and the regulation of the vascular tone are mediated by ion channels (see Fig. 2). Of note, many typical EC mechanosensitive ion channels are also identified in the VSMC, but the functional interaction of them is only sparsely described.

Here, some examples of mechanosensitive ion channels are described which are expressed in different cell layers of the vessel and seem to interact to maintain signal transduction and function within the vessel.

In EC, the mechanosensitive *ENaC* plays a crucial role in the orchestrated mechanism of vascular tone control. The plasma membrane insertion of the channel leads to stiffening of the endothelial surface which is mechanistically linked to the polymerization of the cortical actin leading to a subsequent reduction of NO release upon stimulation with shear stress [49]. In VSMC, ENaC is part of the transduction pathway of constriction response to pressure and acts as potential mechanosensor as it mediates pressure-induced vasoconstriction [41, 89]. Constitutive absence of the endothelial αENaC subunit leads to drastically decreased flow-dependent dilation of mouse mesenteric arteries, indicating that ENaC acts as mechanosensor [167]. Mutations in endothelial β- and γENaC contribute to severe forms of arterial hypertension [83]. Whether VSMC ENaC plays a role in this situation is not known yet. However, the presence of the channel in both cell types and similar regulatory mechanisms [185] let us assume a concerted action in the control of blood vessel tone.

Another example of a mechanosensitive ion channel which is expressed in both EC and VSMC is *Piezo1*. This non-selective cation channel is activated by mechanical stimuli, such as membrane stretch or shear stress. In EC, Piezo1 is activated by shear stress and leads to Ca^2+^ influx and phosphorylation of AKT and eNOS which results in an increased NO production and subsequent VSMC-mediated vasodilation [183]. In contrast, in VSMC, Piezo1 is activated by stretch and involved in processes of vascular remodeling under pathological conditions leading to a decrease in vessel diameter [122, 145]. Together, both Piezo1-dependent mechanisms effectively maintain basal blood pressure regulation.

As mentioned before, many members of the *TRP channel* family are also expressed in both EC and VSMC. In the vascular endothelium, TRP channels are known to act as stretch mechanosensors and to be involved in Ca^2+^ signaling. In VSMC, Ca^2+^ influx through TRP channels in general leads to membrane depolarization. Hence, they play a role in myogenic tone response and vasoconstriction. If and how TRP channels in EC and VSMC do interact is not really resolved at the moment.

In general, there is increasing evidence that the communication between EC and VSMC is not “one-way” from the endothelium to the muscle cells but rather a mutual interaction between both layers. However, shear- or stretch-induced responses in VSMC-free capillaries depend on the mechanosensing by the endothelial cell layer, while the pressure-dependent myogenic response can be attributed to the VSMC.

Recently, myoendothelial junctions have been identified as morphologically distinct structures which are formed by the membranes of both EC and VSMC and appropriate gap junctions between them. These gap junctions are composed of two connexons, composed of at least six connexin proteins. They basically serve as signaling microdomains to enable cross talk between EC and VSMC. Dilating substances, such as NO, are delivered from the EC to the VSMC, whereas IP3 diffusion from VSMC to EC provokes a Ca^2+^-response and leads to constriction. The latter pathway most likely activates intracellular Ca^2+^ stores through TRPV4 (for review see [164]). Thus, via myoendothelial junctions, the cross talk between the endothelium and smooth muscle is facilitated.

In an elegant study by Chiu et al., it was demonstrated that vascular EC function is influenced by the neighboring VSMC. In a co-culture shear stress model, the alignment of EC under flow occurs more rapidly than under static conditions. Furthermore, they conclude that shear stress may lead to a down-regulation of pathophysiological relevant genes and thus may exert vasculoprotective effects [23]. This again is a strong indicator of the functional and physiological relevant interaction between the vascular layers, maintaining vascular tone and reactivity.

## Conclusion and perspectives

Proper regulation of the vessel tone is the basis of cardiovascular health. One of the major mechanisms which contribute to the fine tuning of vasodilation, or contraction is the sensing of mechanical stresses exerted on the vessel wall. In particular, endothelial cells immediately react with a change of their nanomechanical properties and conduct biochemical and/or mechanical signals to the vascular smooth muscle cells. Only the close interaction between all layers of the vascular wall (i) glycocalyx on top of endothelial cells, (ii) endothelial cells, and (iii) smooth muscle cells can maintain vessel tonus, regulate the expression of genes and proteins, and can cause morphological changes. Important mediators of the mechanosignaling are mechanosensitive ion channels expressed in both cell types. Disruption of these ion channel-mediated mechanisms may cause various diseases, such as hypertension and atherosclerosis, commonly described as channelopathies. Gain-of-function mutations in ENaC, for example, lead to a sustained stiffening of the endothelial cell cortex which might contribute to the severe hypertension in patients and mice [83].

There is evidence that TRP channels also contribute to the pathogenesis of hypertension, and it is reported that mutations in TRPC channel genes can be linked to cardiovascular diseases [127]. Expression of TRPC3 for example is elevated in patients with malignant hypertension in the vascular endothelium [169]. TRPM4 may be also involved in the control of blood pressure as TRPM4-deficient mice showed a hypertensive phenotype [116]. In this context, Keiji Naruse introduced the term “mechanomedicine.” This includes the investigation and characterization, but also the therapeutical benefit of this knowledge [124]. Especially, in the cardiovascular system, the mechanosensitive structures could serve as both predictors and pharmaceutical targets in cardiovascular pathologies.
